# Clinical and genetic characteristics of Chinese patients with Shwachman Diamond syndrome: a literature review of Chinese publication

**DOI:** 10.3389/ebm.2024.10035

**Published:** 2024-04-08

**Authors:** Lijun Wang, Youpeng Jin, Yuan Chen, Ping Zhao, Xiaohong Shang, Haiyan Liu, Lifeng Sun

**Affiliations:** Department of Pediatrics, Shandong Provincial Hospital Affiliated to Shandong First Medical University, Jinan, China

**Keywords:** child, clinical presentation, diarrhea, gene pathogenic variant, hematological abnormality

## Abstract

Shwachman Diamond syndrome (SDS) is a rare autosomal recessive genetic disorder and due to its complex and varied clinical manifestations, diagnosis is often delayed. The purpose of this study was to investigate the clinical manifestations and genetic characteristics of SDS in Chinese patients, in order to increase pediatricians’ awareness of SDS and to allow early diagnosis. We conducted a search to identify patients presenting SBDS gene pathogenic variant in two Chinese academic databases. We analyzed and summarized the epidemiology, clinical features, gene pathogenic variants, and key points in the diagnosis and treatment of SDS. We reviewed the clinical data of 39 children with SDS from previously published articles. The interval from the onset of the first symptoms to diagnosis was very long for most of our patients. The age of presentation ranged from 1 day to 10 years (median: 3 months). However, the age of diagnosis was significantly delayed, ranging from 1 month to 14 years (median: 14 months). Hematological abnormalities were the most common presentation, 89.7% (35/39) at the beginning and 94.9% (37/39) at diagnosis of SDS. Diarrhea was the second most common clinical abnormality at the time of diagnosis. 59% (23/39) of patients had a typical history of persistent chronic diarrhea. Furthermore, hepatic enlargement or elevation of transaminase occurred in 15 cases (38.5%). 56.4% patients (22/39) had a short stature, and 17.9% (7/39) patients showed developmental delay. Additionally, twenty patients had compound heterozygous pathogenic variants of c.258 + 2T > C and c.183_ 184TA > CT. Children with SDS in China had high incidence rates of chronic diarrhea, cytopenia, short stature, and liver damage. Furthermore, SBDS c.258 + 2T > C and c.183_ 184TA > CT were the most common pathogenic variants in patients with SDS. The diagnosis of SDS can be delayed if the clinical phenotype is not recognized by the health care provider.

## Impact statement

Shwachman Diamond syndrome (SDS) is a rare autosomal recessive genetic disorder and due to its complex and varied clinical manifestations, diagnosis is often delayed. This study investigated the clinical manifestations and genetic characteristics of SDS in Chinese patients to increase health care provider’s awareness of SDS and to allow early diagnosis.

## Introduction

Shwachman Diamond syndrome (SDS) is an autosomal recessive genetic disease. Common clinical features of SDS are exocrine pancreatic dysfunction, bone marrow failure, congenital abnormalities, and susceptibility to myelodysplastic disorders (MDS) and leukemia, especially acute myeloid leukemia (AML). SDS was first described in the 1960 s and its estimated incidence is 1:77,000 to 1:100,000 [1–3]. This condition is rarely identified in adult patients [3, 4].

As this disease is rarely encountered in the clinic, the reported incidence rate in Europe and the United States is only approximately 0.5/100 000–1.5/100 000 [4–6]. The related literature mostly reports individual case or sporadic small samples. The incidence of SDS is low and clinical understanding is insufficient. In addition, some patients show atypical symptoms. Therefore, it is often misdiagnosed or mismanaged, leading to disease progression and/or life-threatening complications. Therefore, to improve the understanding and diagnosis of SDS in Chinese children, it is necessary to comprehensively summarize cases available in the literatures. Herein, we discuss the presentation and characteristic features of SDS over the past 11 years based on data of 39 patients from two Chinese academic databases. We identified a variety of clinical manifestations of SDS in Chinese children, providing clues for the diagnosis of SDS.

## Materials and methods

We performed a literature search in two Chinese academic databases, namely Wan Fang Medical Online and the China National Knowledge Infrastructure for articles published from January 2010 to April 2021, using the keywords “Shwachman Diamond syndrome,” “SDS,” and “SBDS” in Chinese. We downloaded and reviewed these papers. Only patients with *SBDS* pathogenic variants confirmed by genetic testing were included in the study. Thirteen studies met the inclusion criteria, and data from a total of 39 patients were included [7–19].

Neutropenia was defined as an absolute neutrophil count (ANC) ≤1.5 × 10^9^/L, hemoglobin (Hb) below the normal age-related range was used to define anemia (children aged 6 months to 6 years at Hb levels less than 110 g/L, and children aged 6–14 years are considered anemic when Hb levels are less than 120 g/L), and thrombocytopenia was defined by a platelet count ≤150 × 10^9^/L with no other obvious cause of thrombocytopenia [20]. If the patient has any of these conditions, it is cytopenia. While pancytopenia means a patient has all of these conditions. Pancreatic insufficiency was confirmed by measuring serum trypsinogen (patients aged <3 years), serum amylase (patients aged >3 years), fecal elastase, or 72 h fecal fat [4, 20]. Developmental delay is determined with a child does not attain developmental milestones as compared to peers from the same population [21].

We conducted a comprehensive statistical analysis of the above data and summarized the characteristics of clinical manifestations and the results of genetic tests.

## Results

In our study, information on 39 SDS patients that was published in articles from two Chinese databases were included. In all cases, genetic testing confirmed the presence of pathogenic variants in *SDS*. There was a slight male predominance, with 22 male (56.4%) and 17 female (43.6%) patients. The male: female ratio was 1.3:1. The age of presentation was 1 day–10 years (median: 3 months). However, the age at diagnosis was significantly delayed, ranging from 1 month to 14 years (median: 14 months). Only 17.9% (7/39) patients showed a classic presentation of diarrhea-associated neutropenia after onset. Most children were misdiagnosed with infectious or allergic diarrhea, periodic granulocytopenia, immune thrombocytopenia (ITP), hemolytic anemia, aplastic anemia, and rheumatoid arthritis at the time of presentation (Table 1).

**TABLE 1:** Initial presentation of SDS patients.

Initial presentation	Number of patients (*n* = 39)
Sex	
Male	22 (56.4%)
Female	17 (43.6%)
Age of presentation	
Youngest age	1 day
Oldest age	10 years
Median age	3 months
Age at diagnosis	
Youngest age	1 day
Oldest age	14 years
Median age	14 months
Hematological abnormalities (first CBC)	
Neutropenia	11 (28.2%)
Anemia	14 (35.9%)
Thrombocytopenia	1 (2.6%)
Neutropenia and anemia	5 (12.8%)
Anemia and thrombocytopenia	1 (2.6%)
Pancytopenia	3 (7.7%)
Fever or infections	15 (38.5%)
Diarrhea	14 (35.9%)
Transaminase elevation	9 (23.1%)
Intellectual disability or growth retardation	2 (5.13%)
Liver, spleen, and lymph nodes were swollen	1 (2.6%)
Hearing impairment	1 (2.6%)
Genu valgum	1 (2.6%)
Dyspnea	1 (2.6%)

### First clinical manifestations

Among the 39 children with SDS, the most common initial presentation was hematological abnormalities (35 cases, 89.7%). Furthermore, 1 (2.6%), 11 (28.2%) and 14 (35.9%) patients had thrombocytopenia, neutropenia, and anemia, respectively. Another 5 (12.8%) had neutropenia and anemia simultaneously. One (2.6%) patient had anemia and thrombocytopenia. In addition, three children had pancytopenia. The second-most common initial presentation was fever or infections. Fifteen (38.5%) patients visited the hospital for blood tests and received antibiotics therapy because of fever or infections. They usually had recurrent respiratory and other infections in the early stages of the disease due to cytopenia, especially neutropenia. Some patients were considered to have an immune deficiency disorder. Less than 40% of patients (14 cases, 35.9%) had chronic steatorrhea at the beginning. Transaminase elevation was the first clinical manifestation in 9 (23.1%) patients. Two patients consulted doctors for intellectual disability or growth retardation. Another three patients were admitted to the hospital for dyspnea, hearing impairment, and genu valgum, respectively.

### Characteristic clinical manifestations

The interval from the onset of the first symptoms to diagnosis was very long for most of our patients, with a median duration of 12 months. More than 15 children were diagnosed >2 years after the initial presentation. The characteristic clinical manifestations of the children at diagnosis are shown in Table 2 and summarized as follows (Table 2).

**TABLE 2:** Characteristic clinical manifestations of SDS at diagnosis.

Clinical manifestations	Number of patients (n = 39)
Hematological abnormalities	
Neutropenia	11 (28.2%)
Anemia	8 (20.5%)
Neutropenia and anemia	10 (25.6%)
Neutropenia and thrombocytopenia	3 (7.7%)
Pancytopenia	5 (12.8%)
Diarrhea	23 (59%)
Pancreatin decrease	11 (28.2%)
Pancreatic fatty infiltration (CT or ultrasound)	16 (41%)
Transaminase elevation	15 (38.5%)
Teeth Anomalies	5 (12.8%)
Skeletal Anomalies	17 (43.6%)
Short stature	22 (56.4%)
Neurological problems	
Developmental delay	7 (17.9%)
Generalized weakness/hypotonia	4 (10.3%)
Myelination is underdeveloped	2 (5.1%)
Convulsive	1 (2.6%)
Cognitive and attention deficits	1(2.6%)
Encephalatrophy	1 (2.6%)
Congenital anomalies	
Atrial septal defect	1 (2.6%)
Patent foramen ovale	2 (5.1%)
Patent ductus arteriosus	1 (2.6%)
Hypospadias	1 (2.6%)
Others	
Arthritis	1 (2.6%)
Transformation	6 (15.4%)
AML	1 (2.6%)
MDS	1 (2.6%)
AA	4 (10.3%)

### Hematologic abnormality at clinical presentation

#### Hematological abnormalities were the most common clinical abnormality at the time of diagnosis

Briefly, 37 (94.9%) patients had hematologic abnormalities at the time of diagnosis. Neutropenia (ANC ≤ 1.5 × 10^9^/L) and anemia occurred in 11 (28.2%) and 8 (20.5%) patients, respectively. No patient presented only thrombocytopenia (PLT ≤ 150 × 10^9^/L). Thirteen patients presented neutropenia together with anemia or thrombocytopenia; five had pancytopenia, four were diagnosed with aplastic anemia (AA), and one patient was diagnosed with AML. A 10-year-old patient initially diagnosed with hypocellular MDS presented neutropenia and mild thrombocytopenia, but there was no history of steatorrhea or failure to thrive or cytopenia.

#### Diarrhea was the second most common clinical abnormality at the time of diagnosis

Overall, 59% (23/39) of the patients had a typical history of persistent chronic diarrhea. Routine stool examination showed steatorrhea, often with fat droplets, but no red blood cells or pus cells. Most the patients had a history of chronic diarrhea in childhood, which chronic diarrhea resolved and recovered spontaneously in a few years. The results of the serum lipase and/or amylase test were reported in 13 cases, and 11 (28.2%) had a significant reduction in pancreatic enzyme levels, and the remaining two cases had no history of diarrhea. Pancreatic lipomatosis was observed by computerized tomography (CT) or ultrasound in 16 patients (41%).

#### Liver function tests

Hepatic enlargement (was assessed by ultrasound) or transaminase elevation occurred in 15 cases (38.5%). None of the cases showed positive test results for serotype B or C hepatopathy with diagnostic values. Transaminase levels were restored to normal after treatment by compound glycyrrhizin.

#### Growth and skeletal abnormalities

Twenty-two (56.4%) patients had short stature, 17 (43.6%) presented with skeletal abnormalities, including metaphyseal chondrodysplasia, osteoporosis/osteomalacia and thoracic cage defects, and 5 (12.8%) patients presented teeth abnormalities such as tooth loss and enamel dysplasia.

#### Neurologic disorder

Seven (17.9%) patients exhibited developmental delay, 4 (10.3%) had general weakness/hypotonia, and 2 (5.1%) had underdeveloped myelination. Additionally, one patient had convulsive, one patient had cognitive and attention deficits, one patient had encephalatrophy.

#### Other anomalies

Some patients showed non-classical presentations. One patient with developmental delay had congenital heart disease (patent ductus arteriosus and patent foramen ovale) and hypospadias. Another patient with developmental delay presented atrial septal defect and patent foramen ovale. Furthermore, one newborn had severe dyspnea after birth. The chest radiograph showed that the rib cage was narrow and that the posterior ribs were obviously bent downward in an arched shape. However, none of the patients had obvious facial features or skin lesions or pigmentation common to other types of inherited bone marrow failure syndrome.

### Genetic testing

Genetic testing was carried out in all 39 patients. 23 patients showed *SBDS* heterozygous of c.258 + 2T > C and c.183_ 184TA>CT, 11 patients had c.258 + 2T > C homozygous pathogenic variants, 3 patients had heterozygous pathogenic variants of c.258 + 2T > C or c.183_ 184TA > CT combined with other pathogenic variants, and one patient had c.8T > C homozygous pathogenic variants. In addition, one patient showed *SRP54* heterozygous pathogenic variants of c.349_351de1 (p.T117de1) (Table 3).

**TABLE 3:** *SBDS* genetic mutations.

*SBDS* mutation		Number of patients (n = 39)
c.183_ 184TA>CT, c.258 + 2T>C	compound heterozygous	20 (51.3%)
c.258 + 2T>C	homozygous	11 (28.2%)
c.183_184TA > CT, c.258+2T > C, 292_295delAAAG	compound heterozygous	3 (7.7%)
c.258+2T > C, c.23A > T	compound heterozygous	1 (2.6%)
c.183_184TA > CT, c.201A > G	compound heterozygous	1 (2.6%)
c.8T>C	homozygous	1 (2.6%)
c.258 + 2T>C, c.634_635insAACATACCTGT, c.637_638delGA	compound heterozygous	1 (2.6%)
c.349_351de1 (p. T117del)^a^	heterozygous^a^	1 (2.6%)

^a^ This is heterozygous mutation of *SRP54* gene. It is inherited in an autosomal recessive manner.

## Discussion

SDS is an autosomal recessive disorder with an incidence of 1:77,000 to 1:100,000 [1]. In 1964, scientists from the United States (Shwachman, Diamond, Oski, and Khaw) and Great Britain (Bodian, Sheldon and Lightwood) reported a series of young patients who developed exocrine pancreatic insufficiency with diarrhea and hematologic abnormalities (especially neutropenia, but also varying degrees of thrombocytopenia and anemia) and subsequently failed to develop normally in infancy [3]. The most prominent symptom in childhood is persistent diarrhea, malnutrition, and growth failure due to fat replacement of pancreatic acinar tissue; however, approximately 50% of patients improve spontaneously with age. In our study, the initial symptom was diarrhea in 14 (35.9%) patients. Only seven (17.9%) patients were >5 years of age at the time of onset. Among the older children, four had no symptoms of diarrhea, but all had anemia or neutropenia. A previous study reported that some patients with SDS only exhibited hematologic abnormalities without any gastrointestinal symptoms [22]. Our study showed that the clinical manifestations of SDS in Chinese children were the same as those in other regions.

Several research groups have studied the hematologic features of patients with SDS [23, 24]. Neutropenia, typically defined as a neutrophil count less than 1.5 × 10^9^/L, is the most common hematologic abnormality and affects 88–100% of patients with SDS. MDS and AML are severe life-threatening complications of SDS [24]. In our study, 29 (74.4%) patients had neutropenia. Four (10.3%) patients were diagnosed with AA. An 8-year-old boy developed AML and another 12-year-old patient developed MDS. Previous research showed that the incidence of AML in SDS ranges from 18% to 36% in individuals over 20–30 years, and most patients showing progression to AML are relatively older [25]. Our results were lower than this range. Many pediatricians do not fully understand SDS, and may not do genetic testing and make a proper diagnosis in a timely manner, which may be the main reason.

SDS pancreatic exocrine deficiency typically occurs during the first year of newborn life and is caused by pancreatic acinar cell reduction and fat infiltration. The main manifestations of pancreatic failure are decreased pancreatic elastase content, decreased fat-soluble vitamin content, and increased fecal fat content leading to diarrhea. All these characteristics can often be observed in patients with SDS. Radiographically, magnetic resonance imaging can show pancreatic lipomas. In our study, 23 (62.2%) patients had a typical history of persistent chronic steatorrhea. Two of these patients (cases 11 and 13) were 5 and 6 years old, respectively, and had a history of chronic diarrhea in childhood. Pancreatic lipomatosis was observed in 16 patients.

Other common anomalies, such as hepatomegaly and abnormal liver biochemical tests, are both observed in younger patients. Studies have found that elevated transaminase levels and liver enlargement would gradually return to normal around 5 years of age, which can pose challenges in differential diagnosis. Skeletal abnormalities often occur in late childhood and can occur in 30–50% of patients, including delayed maturation, metaphyseal chondrodysplasia of the long bones, and thoracic abnormalities with thickening of the costal cartilage. Short stature was observed in 22 (56.4%) patients in our study. Some patients had osteoporosis, enamel dysplasia, or cartilage with metaphyseal dysplasia. Some patients were found to have neurological, learning, and/or behavioral disorders [26]. Children with SDS can have significant limitations in academic performance, advanced language skills, intellectual reasoning and perception, including reasoning and visual motor skills [27]. Developmental delay; generalized weakness/hypotonia; underdeveloped myelination; convulsive, cognitive, and attention deficits; and encephalatrophy were found in our study. In addition, some congenital anomalies were presented in our study. One patient had patent foramen ovale, patent arteriosus ductus, and hypospadias. Another patient had atrial septal defect and patent foramen ovale. A wide variety of congenital abnormalities involving the heart, gastrointestinal tract, kidneys, nerves, urinary system, and other organ systems have previously been reported. However, no studies have yet demonstrated that these congenital abnormalities are specific manifestations of SDS.

**FIGURE 1:**
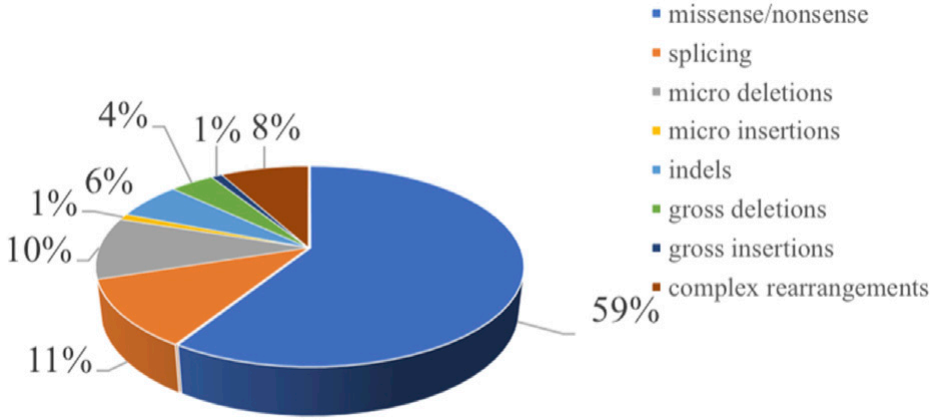
Distribution of the mutation spectrum in SBDS reported in HGMD. A total of 97 mutations in SBDS gene including 59% missense/nonsense mutations, II% splicing, 10% micro deletions, 1% micro insertion, 6% mmicro indels, and 4% gross deletions, 1% gross insertions, and 8% complex rearrangements. The mutations in our patients were all documented by HGMD. Missense mutation/nonsense mutation means the codon encoding an amino acid is replaced by a codon of another amino acid by a base, resulting in a change in the amino acid type and sequence of the polypeptide chain. Splicing is a type of mutation that changes the splicing mode of RNA precursors due to mutations in the splicing donor or acceptor sites or conserved sequences on their sides, so that the resulting mature RNA contains introns or missing exon sequences. Micro deletion/insertion refers to the deletion or insertion of a certain point base of the DNA molecule, causing a change in the reading framework, resulting in a series of downstream code changes, so that the original gene encoding a peptide chain into another completely different peptide chain sequence. Insertion—deletion mutations (indels) refer to insertion and/or deletion of nucleotides into genomic DNA and include events less than I kb in length. Gross deletion/insertion means single exon or multiple exons missing/inserted. Complex rearrangements refers to repetitive replication, inversion, translocation, and inter-chromosome trans-location occur in a region within the chromosome. Usually refers to the deletion, insertion, duplication, inversion, translocation, and change in copy number variants (CNVs) of DNA fragments larger than 1 kb in the genome.

Chromosome 7q11 plays an important role in bone marrow cell proliferation, mitosis, and the matrix microenvironment, and more than 90% of patients with SDS carry biallelic pathogenic variants in the *SBDS* gene on chromosome 7q11 [1]. We searched for gene pathogenic variants reported in both cases and literature of *SBDS* in the Human Gene Mutation Database (HGMD^®^). A total of 97 pathogenic variants in the *SBDS* gene were reported and included 59% missense/nonsense pathogenic variants, 11% splicing events, 10% small deletions, 1% small insertion, 6% small indel, and 4% gross deletions, 1% gross insertions, and 8% complex rearrangements (Figure 1). The most common genetic pathogenic variants were c.258 + 2T > C and c.183_ 184TA > CT [28, 29]. Similarly, in our study, 20 patients had heterozygous pathogenic variants of c.258 + 2T > C and c.183_ 184TA > CT. Eleven patients harbored c.258 + 2T > C homozygous pathogenic variants, while 7 patients presented heterozygous pathogenic variants of c.258 + 2T > C or c.183_ 184TA > CT combined with other pathogenic variants. Though the identification of *SBDS* in 2003, *DNAJC21*, *EFL1,* and *SRP54* genes also have been reported causal SDS. In our patients, there was one patient showed *SRP54* heterozygous pathogenic variants. Approximately 90% of patients with typical presentation have pathogenic variants in the *SBDS* gene, but 10% of patients with clinically diagnosed SDS may not have any known pathogenic variant. However, it remains unclear whether these *SBDS* pathogenic variant-negative patients represent different genetic variations in SDS. Therefore, the limitation of this study is that only *SBDS* pathogenic variant-positive SDS were included. Additional clinical and molecular studies are needed to further characterize *SBDS* pathogenic variant-negative patients.

All the clinical features of SDS can occur during childhood and adolescence. Therefore, the clinical diagnosis is usually established in children and only sometimes in adults. *SDBS* genotyping should always be performed for confirmation. The early diagnosis of SDS is challenging, especially in the neonatal period. Through a multidisciplinary approach to the prevention and treatment of symptoms, the early diagnosis of SDS may improve the overall healthcare of patients. The early diagnosis of SDS is crucial to monitor the risk and clinical symptoms of hematologic malignancies. To avoid delayed treatment and to ensure timely detection of the risk of developing MDS or AML, children with recurrent liver dysfunction with unknown etiology and failure to thrive are recommended early genetic testing of SDS [30].

To date, no specific treatment for SDS has been established. Therefore, a multidisciplinary approach to SDS management may be useful. For gastrointestinal manifestations, the primary treatment is pancreatic enzyme therapy, medium chain triglycerides, and fat-soluble vitamin supplements, along with a normal to high-fat diet. Although most SDS-positive individuals present some hematological manifestations of their disease, most do not require transplantation. In SDS, the estimated risk of the development of myelodysplastic syndrome and AML is 19% at 20 years and 36% at 30 years [24]. Recent studies have indicated that for patients with SDS who develop BMF or convert to myeloid malignancy, hematopoietic stem cell transplantation is the only curative approach for the disease. However, transplantation does not prolong the survival of those children. Therefore, further research is needed to improve patient outcomes.

## Conclusion

Our study reveals a wide range of clinical presentations for SDS. Analysis of Chinese patients showed that children with SDS in China had high incidence rates of chronic diarrhea, cytopenia, short stature, and liver damage. Furthermore, the most common genetic pathogenic variants in patients with SDS were *SBDS* c.258 + 2T > C and c.183_ 184TA > CT. The diagnosis of SDS can be delayed if the clinical phenotype is not recognized by the health care provider.

## Data Availability

The original contributions presented in the study are included in the article/Supplementary material, further inquiries can be directed to the corresponding authors.
